# Repeated open endotracheal suctioning causes gradual desaturation but does not exacerbate lung injury compared to closed endotracheal suctioning in a rabbit model of ARDS

**DOI:** 10.1186/1471-2253-13-47

**Published:** 2013-12-05

**Authors:** Hideaki Sakuramoto, Nobutake Shimojo, Subrina Jesmin, Takeshi Unoki, Junko Kamiyama, Masami Oki, Ken Miya, Satoru Kawano, Taro Mizutani

**Affiliations:** 1 Department of Emergency and Critical Care Medicine, Faculty of Medicine, University of Tsukuba, Tsukuba, Ibaraki 305-8575, Japan

**Keywords:** Acute respiratory distress syndrome, Lung injury, Repeated endotracheal suctioning, Repeated derecruitments, Mechanical ventilation

## Abstract

**Background:**

Although endotracheal suctioning induces alveolar derecruitment during mechanical ventilation, it is not clear whether repeated endotracheal suctioning exacerbates lung injuries. The present study aimed to determine whether repeated open endotracheal suctioning (OS) exacerbates lung injury compared to closed endotracheal suctioning (CS) during mechanical ventilation in an animal model of acute respiratory distress syndrome (ARDS).

**Methods:**

Briefly, thirty six Japanese white rabbits were initially ventilated in pressure-controlled mode with a constant tidal volume (6 mL/kg). Then, lung injury was induced by repeated saline lavage. The rabbits were divided into four groups, namely: a) OS; b) CS; c) control with ARDS only; d) and healthy control (HC) without ARDS. Animals in all the groups were then ventilated with positive end expiratory pressure (PEEP) at 10 cm H_2_O. CS was performed using 6 French-closed suctioning catheters connected to endotracheal tube under the following conditions: a) a suctioning time and pressure of 10 sec and 140 mm Hg, respectively; and b) a suction depth of 2 cm (length of adapter) plus tracheal tube. OS was performed using the same conditions described for CS, except the ventilator was disconnected from the animals. Each endotracheal suctioning was performed at an interval of 30 min.

**Results:**

PaO2/FIO2 (P/F) ratio for CS, control and HC groups remained at >400 for 6 hours, whereas that of the OS group progressively declined to 300 (*p* < 0.05), with each suctioning. However, no difference was observed either in lung injury score (histology) or in the expression pattern of inflammatory cytokines (tumor necrosis factor-α and interleukin-6) after 6 hours between the OS and CS groups in the circulatory as well as the pulmonary tissues.

**Conclusions:**

Progressive arterial desaturation under conditions of repeated endotracheal suctioning is greater in OS than in CS time-dependently. However, OS does not exacerbate lung injury during mechanical ventilation when observed over a longer time span (6 hours) of repeated endotracheal suctioning, based on morphological and molecular analysis.

## Background

Acute respiratory distress syndrome (ARDS) is one of the most challenging problems in critical care medicine, with substantial mortality and significant long-term morbidity [1]. Mechanical ventilation is a life-saving tool for patients with ARDS. However, as with any therapy, it also has the potential to cause or aggravate progressive tissue damage or lung injury, a phenomenon often referred to as ventilator–induced lung injury (VILI) [2,3]. VILI is characterized by vascular leakage and inflammatory responses that ultimately lead to pulmonary dysfunction [4]. VILI is now considered as one of the most serious complications of mechanical ventilation and involves several mechanisms [5], namely: alveolar over-distension (volutrauma), atelectrauma, barotrauma and inflammatory reactions (biotrauma). Concerning volutrauma, a previous study demonstrated an improvement in the survival rate of patients with ARDS using low tidal volume ventilation [6]. Atelectrauma is created by tidal cyclic openings and closure of collapsed alveoli, also called repeated derecruitments. Repeated derecruitment of previously recruited lungs can exacerbate lung injuries during mechanical ventilation [7,8]. Such inflicted injuries may subsequently stimulate a cascade of biological responses, leading to further lung injury (biotrauma) [9,10]. Importantly, biotrauma will not only aggravate ongoing lung injury, but can also lead to multiple organ failure. The key to a successful clinical management of patients with ARDS is preventing further advancement of VILI. For this reason, the main goal of the latest strategies for lung protective ventilation has been prevention of alveolar over-distension and derecruitment.

In order to achieve optimal alveolar recruitment, patients with ARDS are often exposed to high levels of positive end expiratory pressure (PEEP). Exposure of ARDS patients to unintended sudden withdrawal of PEEP (due to transport of patients, alternating PEEP, endotracheal suctioning, etc.) may aggravate lung injury/collapse and decrease oxygenation. Although endotracheal suctioning is known to be one of the causes of repeated derecruitments during mechanical ventilation, it is still routinely performed in patients with ARDS. More recently, a growing body of evidence demonstrates discretely the difference of open endotracheal suctioning (OS) and closed endotracheal suctioning (CS) on the respiratory and hemodynamic parameters in ARDS subjects/models during mechanical ventilation. The reports suggest that OS induces alveolar derecruitment [11]. In the presence of ARDS, the massive loss of lung volume induced by the disconnection of the patient from the ventilator is the predominant mechanism of hypoxemia [12]. Furthermore, the high negative suctioning pressure required for removing bronchial secretions contributes to the loss of lung volume. In contrast, CS is effective to prevent alveolar derecruitment by avoiding ventilator disconnection, thereby maintainig appropriate oxygenation [11]. On the other hand, a previous study reported that CS also causes desaturation and derecruitment during mechanical ventilation in pediatric patients [13]. The short term effects of endotracheal suctioning are clear (i.e., desaturation and loss of lung volume), but long term and repetitive effects, especially lung injury or molecular alternations, are not clear. Thus, it is unclear whether repeated endotracheal suctioning can exacerbate lung injuries during mechanical ventilation. Additionally, no study to date has investigated the effects of repeated OS vs. repeated CS on: a) lung morphology and molecular profile of crucial cytokines at the circulatory and pulmonary tissue levels; and b) the profile of hemodynamic and respiratory parameters in lavage-induced surfactant depleted ARDS models during mechanical ventilation.

The facts stated above led us to hypothesize that repeated endotracheal suctioning, especially open suctioning of longer time span, could cause continuous alveolar derecruitment, resulting in gradual arterial desaturation and, subsequently, exacerbate lung injury with atelectrauma. The aim of the present study was to assess whether repeated derecruitments induced by OS exacerbates lung injury compared to CS during mechanical ventilation with high PEEP in lavage-induced surfactant, depleted ARDS models. It is anticipated that data generated from this present study will clarify the effects of repeated OS vs. CS on VILI.

## Methods

### Animal preparation and measurements

Thirty six male Japanese White rabbits weighing between 2.8 and 3.5 kg were anesthetized using sodium pentobarbital (75–150 mg, bolus infusion) and restrained in a supine position. Under local anesthesia using 1.0% lidocain solution (0.25 mg/kg), the ventral side of the neck was carefully dissected and a tracheostomy was performed, and an endotracheal tube (3.5 mm internal diameter) placed in the trachea and tied in order to stabilize it. The animals were then ventilated with a LTV-1000 ventilator (CareFusion, San Diego, CA) in pressure-controlled mode with PEEP of 2 cm H_2_O, inspiratory time of 0.5 sec and inspired oxygen fraction of 1.0. Airway pressure was adjusted constantly to achieve constant expiratory tidal volume of 6 mL/kg. Initial respiratory rate was set to achieve normo-carbia. Mechanical ventilation was continued in the same manner throughout the experiment, except for the adjustments of PEEP level described later. Anesthesia and muscle paralysis were maintained by continuous infusion of sodium pentobarbital (5 mg/kg/h) and pancuronium (0.1 mg/kg/h) via infusion pump through the ear vein. Normal saline (3 mL/kg/h) was then continuously infused as maintenance fluid. The right carotid artery was catheterized for blood gas sampling and monitoring of arterial pressure. Heart rate and mean arterial pressure were monitored using Philips IntelliVue MP50 Patient Monitor (Philips Medizin Systeme GmbH, Böblingen, Germany). Body temperature was monitored continuously using a rectal probe and was maintained between 38 and 39°C using a heating pad.

Arterial blood gases were measured with blood drawn from the carotid artery using an ABL 720 blood gas analyzer (Radiometer Copenhagen, Copenhagen, Denmark). Expiratory tidal volume and airway pressures were recorded from the ventilator display. Effective tidal volume was calculated by subtracting the compression volume of the ventilator circuit from the tidal volume. The animal protocol of the present study was approved by the Ethics Committee of the Animal Resource Center of the University of Tsukuba. The animals were cared for in accordance with the guidelines for ethical animal research.

The experimental animals were divided into four groups, namely: a) OS with ARDS (OS); b) CS with ARDS (CS); c) a control group with ARDS, but without endotracheal suctioning (Control); d) and a healthy control group with 6 hours of ventilation, but without ARDS and endotracheal suctioning (HC). Animals in the control and HC groups were, however, not randomly assigned to their respective groups. In order to check and validate the results of the present study all the experiments were repeated using newly added control and HC groups.

### Induction of lung injury

After 30 min of stabilization, baseline data were recorded. Through the endotracheal tube, 15 mL/kg of normal saline solution at 38°C was administered into the lung, using a modification of the technique described earlier by Lachmann et al. [14]. After instillation was completed, rabbits were mechanically ventilated with a pressure not exceeding 28 cm H_2_O for a minute or until severe bradycardia (<40 beats/min). The animals were gently rotated from side to side in order to help spread saline solution uniformly. Saline solution was drained out of the lung by gravity and then actively suctioned with a suction catheter. After the first lavage, and between subsequent lavages, the animals were ventilated for 5 min with a peak inspiratory pressure (PIP) of 12 cm H_2_O, a PEEP of 2 cm H_2_O. Arterial blood (0.4 mL) was then sampled for blood gas analysis. Lavage was repeated until the arterial blood gas, drawn 5 min later, showed PaO_2_/FIO_2_ ratio (P/F) < 100. After confirmation of a stable severe lung injury by performing another arterial blood gas 30 min later (P/F <100), the experimental protocol was begun, as described below.

### Ventilation protocols

After lung injury, intermittent mandatory pressure control ventilation was set as follows: a) the fraction of inspired oxygen was set at 1.0; b) tidal volume was set at 6 mL/kg, c) inspiratory time was at 0.5 sec, d) PEEP was set at 10 cm H_2_O, e) the mandatory respiratory rate was set at 30/min and f) the inspiratory pressure limit was set at 28 cm H_2_O (the PIP was limited to 28 cm H_2_O in order to prevent early deaths from pneumothorax, which occurred in most animals during a pilot study when higher PIP values were used). The mandatory respiratory rate was subsequently adjusted to maintain the PaCO_2_ in the range of 60–100 mm Hg, where possible, with a rate of 30 - 40/min [15].

### Endotracheal suctioning protocols

After lung injury, CS was performed twice every 30 minutes during ventilation, using a 6 French-closed suctioning catheter system (Trachcare, Ballard Medical products, Draper, UT), which was connected to the endotracheal tube under the following conditions: a) Suctioning time and pressure of 10 sec and 140 mm Hg (20 Kpa), respectively; and b) Suction depth of 2 cm (length of adapter) plus length of tracheal tube [16]. OS was performed with the same catheter (Trachcare) under the same conditions, except with a disconnected ventilator circuit from the animal. After OS, ventilator circuit was reconnected at the previous settings. All data were collected at baseline, at injury, and hourly just before suctioning for a total of 6 h. After completion of the 6 h ventilation, animals were killed with bolus injections of sodium pentobarbital (50 mg/kg). The left lung was rapidly removed and snap-frozen in dry ice.

### Expression levels of potential inflammatory cytokines as revealed by Enzyme-Linked Immunosorbent Assay (ELISA) and Real Time PCR

The concentrations of selected inflammatory cytokines, namely, interleukin (IL) -6 and tumor necrosis factor (TNF) -α, in lung tissue and serum at 6 h ventilation were determined using rabbit specific commercial ELISA kits (USCN Life Science & Technology, Missouri City, TX).

The mRNA expression of IL-6 and TNF-α were assessed by Real Time PCR. Total RNA was isolated using an RNA purification kit (Qiagen, Hilden, Germany) and was used for PCR assay to detect mRNA expression. Reverse transcription (RT) of total RNA (2 μg) was performed in a final volume of 100 μl containing 1 × TaqMan RT buffer, 5.5 mM MgCl_2_, 500 mM/L each deoxy-unspecified nucleoside 5’-triphsophate, 2.5 mM random hexamers, 0.4 U/μl RNase inhibitor, and 1.25 U/μl multiscribe RT. The action mixture was covered and amplification was initiated by 1 min denaturation at 95°C for 1 cycle, followed by multiple (45 – 50) cycles at 95°C for 15 sec and 60°C for 60 sec using a Lightcycler 480 PCR system (Roche Applied Science). Real Time PCR were carried out as described [17], using rabbit specific TaqMan kits Applied Biosystems, assay-ID Oc04097053_m1 for IL-6 mRNA, Oc03397715_m1 for TNF-α mRNA and Oc03823402_g1 for Glyceraldehyde-3-phosphate dehydrogenase (GAPDH) mRNA. For internal control, GAPDH was used.

### Histological analysis

The right lungs were inflated with 4% formaldehyde at a pressure of 20 cm H_2_O via trachea and were fixed in 4% formaldehyde for >24 h. Subsequently the lungs were divided into 4 regions with a #11 blade scalpel. Each region was then sectioned, stained with hematoxylin and eosin and scored by two investigators blinded to experimental conditions. Samples were assigned an injury score in each of the 5 categories (edema, hemorrhage, neutrophil infiltration, bronchiolar epithelial desquamation, and hyaline membrane formation) based on severity (0 = not present, 4 = severe and present throughout), as previously described [18,19]. Regional composite lung injury scores were calculated by summing the category scores within each lung region. Whole lung injury scores were calculated by summing the regional composite lung scores within each animal.

### Statistical analysis

Baseline variables and the mRNA expression were expressed as mean ± SD. Intra-intergroup differences were compared by one way analysis of variance adjusted Bonferroni’s. Hemodynamic and gas exchange variables were expressed as mean ± SD. Repeated-measures analysis of variance was used to determine intra group differences. Specific intergroup differences and time points of this difference were determined by using Bonferroni’s correction for multiple comparisons. Lung injury score and cytokine concentrations were expressed as medians and interquartile range (25th and 75th percentiles) and the data were analyzed using Kruskal wallis one way analysis of variance. The data from each group were compared with the previous time point starting from baseline injury by a test of within-subjects differences of repeated-measures analysis of variance by IBM-SPSS version 19.0 software (IBM-SPSS Inc., Chicago, IL).

## Results

### Baseline characteristics

Baseline characteristics of the animals in the study groups are shown in Table 1. There were no differences in body weight, hemodynamic variables and gas exchange parameters before the induction of lung injury.

**Table 1:** Baseline characteristics

	**OS group**	**CS group**	**Control group**	**HC group**	***p *** **value**
	**n = 13**	**n = 13**	**n = 7**	**n = 3**	
Body weight, kg	3.1 ± 0.3	3.0 ± 0.3	2.8 ± 0.2	3.0 ± 0.2	0.169
Lavage, times	2.9 ± 0.5	3.0 ± 0.6	3.0 ± 0.6	Non	0.929
MAP, mmHg	131 ± 16	118 ± 13	116 ± 7	119 ± 2	0.063
HR, beats /min	236 ± 65	217 ± 48	280 ± 68	213 ± 183	0.349
RR, breaths /min	23.5 ± 5.6	22.7 ± 6.2	22.3 ± 2.7	24.0 ± 1.0	0.950
P/F ratio	460 ± 51	477 ± 54	427 ± 36	412 ± 38	0.085
PaCO_2_, mmHg	44.4 ± 4.6	46.1 ± 5.5	40.6 ± 10.2	44.5 ± 4.3	0.363

OS, open endotracheal suctioning; CS closed endotracheal suctioning; HC healthy control; MAP, mean arterial pressure; HR, heart rate; RR, respiration rate; P/F ratio, PaO_2_/FIO_2_ ratio. Values are mean **±** SD.

### Gas exchange

**Figure 1:**
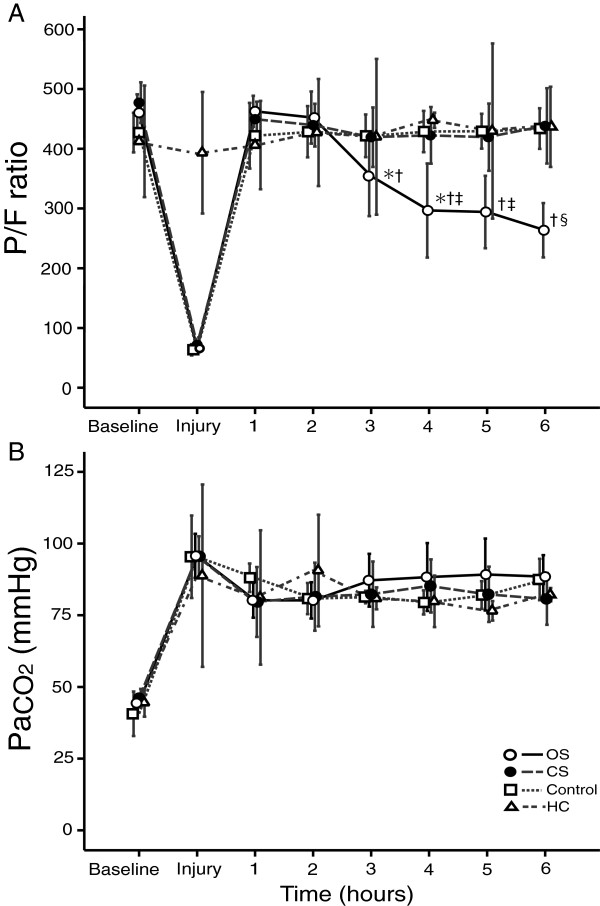
**Changes in (A) PaO**_**2**_**/FIO**_**2 **_**(P/F) ratio and (B) PaCO**_**2 **_**in the study groups.** OS, open endotracheal suctioning (*open circle*); CS, closed endotracheal suctioning (*closed circle*); Control, control group with ARDS, but without endotracheal suctioning (*square*); HC, healthy control group with 6 hour ventilation, but without ARDS and endotracheal suctioning (*triangle*). **(A)** OS group shows progressive decline in P/F, whereas, all other groups maintained at P/F of >400 up to the end of the study. *p < 0.05 vs. compared with previous value within the same group; ^†^*p* < 0.05 compared with 1 hour after injury within the same group; ^‡^*p* < 0.05 vs. CS and Control groups; ^§^*p* < 0.05 vs. CS, Control and HC groups. Data are shown as means with 95% confidence intervals.

After lung injury was induced, P/F ratio was reduced to a mean of 63 ± 13, 73 ± 20 and 64 ± 9 for the CS, OS and Control groups, respectively (*p* = 0.511). After PEEP levels were increased to 10 cm H_2_O, P/F increased to >400 in all groups (Figure 1A). In the CS, control and HC groups, P/F remained at 400 throughout the study period. However, in the OS group, P/F decreased continuously and dropped to a mean of 297 ± 124 at 4 h, to 294 ± 95 at 5 h and to 264 ± 71 at 6 h (all *p* = 0.000 vs. P/F at 1 h after injury). This P/F level was significantly lower than in the CS groups (*p* = 0.013, *p* = 0.005 and *p* = 0.000 at 4, 5 and 6 h, respectively) (Figure 1A).

At injury, PaCO_2_ for all groups increased significantly (Figure 1B) compared to the baseline level. Overall, PaCO_2_, pH and serum lactate levels did not differ significantly among all groups at baseline and throughout the 6 h study period (Figure 1B and Table 2). PIP significantly increased after the increase in PEEP to 10 cm H_2_O, compared with baseline levels. PIP levels were significantly higher than those of the HC group in the OS, CS and control groups after injury. Thereafter, PIP showed a similar trend for the duration of the 3 h after injury. However, in the OS group PIP levels were significantly higher than in the other groups at the 4, 5 and 6 h post-injury interval (Table 2).

**Table 2:** Sequential changes in variables of lung mechanics and hemodynamics

**Variables**	**Group**	**Baseline**	**Injury**	**1 Hr**	**2 Hr**	**3 Hr**	**4 Hr**	**5 Hr**	**6 Hr**
PIP, cmH_2_O	OS	13.0 ± 3.0	19.3 ± 3.0^*a*^	22.6 ± 2.1^*ad*^	23.0 ± 2.6	23.0 ± 2.1	23.8 ± 2.1	24.6 ± 2.6 ^*b*^	24.9 ± 2.9 ^*b*^
	CS	13.2 ± 1.7	19.4 ± 2.6^*a*^	21.2 ± 1.6 ^*d*^	21.3 ± 2.3	21.3 ± 2.3	21.5 ± 1.9 ^*c*^	21.5 ± 2.2 ^*c*^	21.8 ± 2.2 ^*c*^
	Control	13.2 ± 2.0	20.5 ± 2.1^*a*^	20.8 ± 1.0^*d*^	21.3 ± 1.0	21.0 ± 1.3	20.3 ± 1.4 ^*c*^	19.8 ± 1.2^*c*^	19.2 ± 1.5 ^*c*^
	HC	13.7 ± 1.5	17.7 ± 0.6	17.3 ± 0.6	19.3 ± 1.2	19.6 ± 2.3	19.3 ± 2.5 ^*c*^	19.0 ± 2.0 ^*c*^	18.3 ± 1.2 ^*c*^
Arterial pH	OS	7.43 ± 0.03	7.05 ± 0.14^*a*^	7.15 ± 0.13^*a*^	7.14 ± 0.13	7.11 ± 0.12	7.09 ± 0.15	7.09 ± 0.17	7.11 ± 0.14
	CS	7.40 ± 0.05	7.11 ± 0.15^*a*^	7.16 ± 0.11	7.15 ± 0.10	7.16 ± 0.14	7.12 ± 0.14	7.14 ± 0.08	7.17 ± 0.09
	Control	7.48 ± 0.08	7.20 ± 0.08^*a*^	7.22 ± 0.06	7.23 ± 0.06	7.26 ± 0.03	7.26 ± 0.05	7.24 ± 0.05	7.22 ± 0.06
	HC	7.41 ± 0.06	7.22 ± 0.04	7.24 ± 0.02	7.23 ± 0.03	7.22 ± 0.03	7.24 ± 0.05	7.24 ± 0.05	7.20 ± 0.04
MAP, mmHg	OS	131 ± 17	118 ± 20	98 ± 14^*a*^	102 ± 16	101 ± 14b	100 ± 20	97 ± 11	95 ± 16
	CS	119 ± 13	121 ± 18	101 ± 14^*a*^	102 ± 12	94 ± 15	94 ± 16	94 ± 16	91 ± 15
	Control	116 ± 7	119 ± 11	93 ± 10 ^*a*^	95 ± 11	96 ± 9	102 ± 13	106 ± 13	105 ± 9
	HC	118 ± 2	111 ± 6	105 ± 13	112 ± 26	100 ± 15	96 ± 6	102 ± 16	105 ± 15
HR, beats/min	OS	241 ± 64	209 ± 31	217 ± 43	215 ± 24	200 ± 19	211 ± 36	211 ± 37	207 ± 31
	CS	214 ± 49	195 ± 29	221 ± 49	218 ± 32	227 ± 43	224 ±49	208 ± 34	212 ± 27
	Control	272 ± 49	242 ± 53	244 ± 30	253 ± 37	252 ± 37^*c*^	243 ± 39	232 ± 39	235 ± 20
	HC	299 ± 38	207 ± 140	231 ± 11	228 ± 24	225 ± 11	253 ± 15	247 ± 45	235 ± 44

OS, open endotracheal suctioning; CS, closed endotracheal suctioning; HC, Healthy control; PIP, peak inspiratory pressure; MAP, mean arterial pressure; HR, heart rate. ^*a*^*p* < 0.05 compared with previous value within the same group; ^*b*^*p* < 0.05 compared with 1 hour after injury within the same group; ^*c*^*p* < 0.05 vs. OS group; ^*d*^*p* < 0.05 vs. HC group. Values are mean **±** SD.

### Hemodynamic variables

Overall, there was no significant difference in mean arterial pressure, heart rate (Table 2) and arterial lactate levels among all groups.

### Histological analysis

**Figure 2:**
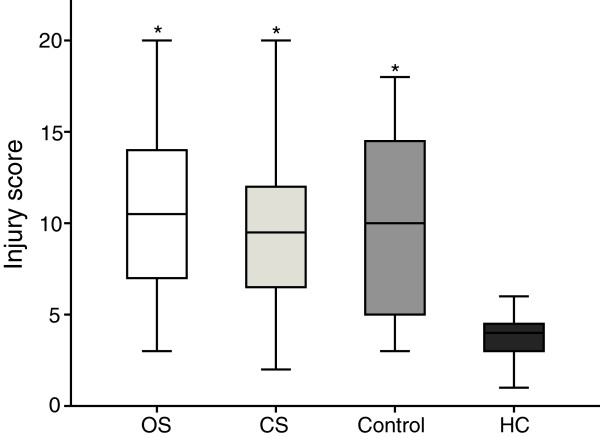
**Box-and-whiskers graph of quantitative histological analysis showing the lung injury score.** The ends of the boxes indicate the 25th and 75th percentiles and the lines in the bars indicate the median value. The 10th and 90th percentiles were indicated with whiskers. OS, open endotracheal suctioning; CS, closed endotracheal suctioning; Control, control group with ARDS, but without endotracheal suctioning; HC, healthy control group with 6 hour ventilation, but without ARDS and endotracheal suctioning. *p < 0.05, compared with healthy control (HC) group.

Histological data were expressed as median (interquartile range). The Lung injury scores were lower in the HC group compared to all other groups (*p* < 0.007). The median values for lung injury scores in the CS, OS, control and HC groups were 10 (7.0 – 14.5), 9.5 (6.0 – 12.0), 10.0 (5.0 – 14.5) and 4.0 (3.0 – 5.0), respectively (Figure 2).

### Expression pattern of IL-6 and TNF-α protein

**Figure 3:**
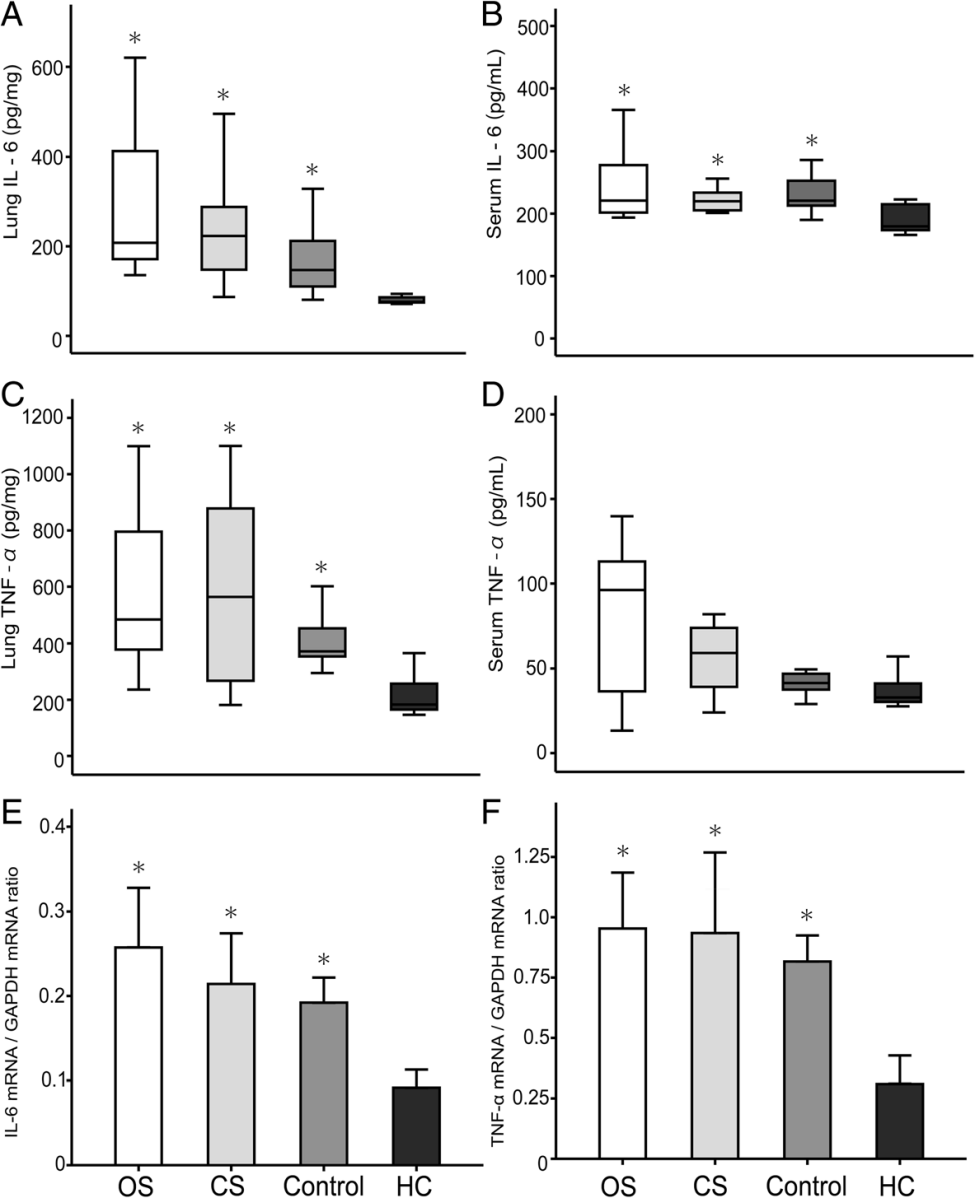
**Expression level of potential inflammatory cytokines as revealed by ELISA and Real Time PCR.** Serum and pulmonary levels of interleukin (IL)-6 and tumor necrosis factor (TNF) –α at the end of the study by ELISA **(A-D)**. The mRNA expression of IL-6 and TNF-α at the end of the study by Real Time PCR **(E-F)**. OS, open endotracheal suctioning; CS, closed endotracheal suctioning; Control, control group with ARDS, but without endotracheal suctioning; HC, healthy control group with 6 hour ventilation, but without ARDS and endotracheal suctioning. *p < 0.05, compared with healthy control (HC) group.

There were no significant differences observed in pulmonary and serum protein concentrations of IL-6 and TNF-α between OS and CS groups, as demonstrated by ELISA (Figure 3). Pulmonary and serum concentrations of IL-6 and pulmonary concentrations of TNF-α were lower in HC group compared to all other groups (*p* < 0.005). The median values for IL-6 pulmonary concentrations (pg/mg) in the CS, OS, control and HC groups were 207 (170–449), 233 (141–294), 147 (96–212) and 75 (74–86), respectively (Figure 3A). IL-6 serum concentrations (pg/mL) in the CS, OS, control and HC groups were 220 (201–281), 219 (205–235), 220 (212–260) and 179 (171–216), respectively (Figure 3B). TNF-α pulmonary concentrations (pg/mg) in the CS, OS, control and HC groups were 485 (348–815), 564 (262–898), 372 (352–489) and 183 (160–287), respectively (Figure 3C). These results were confirmed and complemented by data generated from mRNA expression (Figure 3). Consistent to IL-6 and TNF-α protein levels, the pulmonary mRNA expression levels of these cytokines were not significantly different between CS and OS groups (Figure 3D and E).

## Discussion

The key findings of the present study are that: a) repeated open endotracheal suctioning causes gradual and time-dependent reductions in arterial oxygenation over the course of endotracheal suctioning; b) repeated derecruitments induced by multiple OS do not exacerbate lung injury, based on evidence from histological analysis using whole lung injury scoring system; c) expression levels of the crucial serum and pulmonary inflammatory cytokines remained unchanged throughout the process of repeated OS compared to CS during mechanical ventilation in an ARDS rabbit model induced by surfactant depletion. This is the first study that uses a longer time course, i.e., intermittent endotracheal suctioning over 6 hours, to investigate the effects of repeated suctioning under a well-controlled experimental setting.

Endotracheal suctioning is the most common secretion management procedure performed in mechanically-ventilated patients, even though lung volume loss, hypoxemia and hemodynamic compromise are known risk factors of such a procedure [11,20-23]. Also, progressive atelectasis in ARDS can exacerbate hypoxemia. In addition, it may produce lung and systemic injuries through the release of cytokines and right-ventricular failure [24]. Our present findings on arterial desaturation are similar to those of other groups that have evaluated CS [11,21,25-27]. Consistent to our results, other groups have also found that arterial desaturation related to endotracheal suctioning is greater with OS than with CS [11,21,25-27]. Taken together, these findings imply that arterial desaturation in ARDS is unaffected by either single or repeated OS. However, it is important to note that while the present study that used 6 h to measure arterial oxygenation, the previous studies only used 10–30 min maximum after endotracheal suctioning [11,21-31]. Therefore, previous studies cannot elucidate whether transient fluctuations in arterial oxygenation occur immediately following endotracheal suctioning and how long the trend in arterial desaturation persisted. Here, we provide the first evidence that repeated OS causes gradual reductions in arterial oxygenation over a prolonged time span of 6 hours. Specifically, we showed in our data statistically significant reduction in arterial oxygenation at 4, 5 and 6 hours of endotracheal suctioning, suggesting a clear time-dependent reduction in arterial oxygen level through repeated OS. For now, the exact mechanism underlying this gradual and time-dependent decrease in oxygenation of the OS group is not clear. It is likely that continuous alveolar derecruitment is responsible for this progressive desaturation.

Indeed, a much greater end-expiratory lung-volume change with OS than with CS has been documented. Specifically, Maggiore et al. [11] found a 123 mL end-expiratory volume change with CS versus a 1645 mL volume change with OS in six ARDS patients, using inductive plethysmography. Brochard et al. [20], using computed tomography, observed a 300 mL end-expiratory lung-volume change during OS versus 100 mL change for CS. Advocates of CS have argued that lung volume recovers more quickly following suctioning [30], possibly accounting for the oxygenation benefits of CS in preterm infants reported in some studies [31]. Tingay et al. [29] found that CS preserves global lung volume only when using a small suction catheter relative to the internal diameter of the endotracheal tube. In the present study, the catheter size was chosen according to the current guidelines [16], in order to simulate clinical settings. The influence of suction catheter size during CS, but not OS, may also explain the variability in previous study results those made comparison between OS and CS in terms of global lung volume, oxygenation and heart rate in newborn infants [30,31]. We believe that our model is more representative of the diseased neonatal lung with regard to chest wall size, illness severity and endotracheal suctioning method. Clinical studies to compare the changes in oxygenation over a prolonged period of time between OS and CS seem to be needed in the future.

Conflicting reports exist concerning the effectiveness of CS in removing secretions compared to OS. Although CS is a safe method of endotracheal suctioning, previous studies reported that CS was less effective than OS in removing secretions [12,32]. Therefore, sometimes there is still a need to perform OS, as well as recruitment maneuver after OS in order to restore lung volumes and to prevent desaturation in clinical settings [11,23]. In addition, recruitment maneuver may prevent VILI to open atelectasis [8,33]. In our study, recruitment maneuver was not performed to evaluate the effects of hyperinflation independently. If recruitment maneuver was performed in our study, OS might not have caused progressive desaturation. However, recruitment maneuver may induce lung stress and strain, which include several factors, such as the level of pressure, time to reach inspiratory pressure and frequency, leading to VILI [33,34].

During mechanical ventilation, repeated derecruitments (induced by alternating PEEP or disconnected from ventilator) of initially recruited lung accentuates lung injury [7,8]. The major site of this injury has been localized at the bronchiolar level [7,8]. However, the effects of repeated endotracheal suctioning during mechanical ventilation in ARDS subjects on the aggravation of further lung injury is yet to be investigated. The present study showed that no significant differences in lung injury score existed in lungs that have already been derecruited, irrespective of repeated endotracheal suctioning, i.e., either open or closed. It is important to note that all the regions of both lungs were carefully and blindly checked morphologically and that no significant difference in injury score was found between the endotracheal suctioning groups. These findings contradict data showing detrimental effects of OS in ARDS subjects that have undergone mechanical ventilation. However, the present findings on OS-induced arterial desaturation are consistent with previous study results [11,21,25-27]. In addition, we believe that the low tidal volume setting in our current experimental protocol during mechanical ventilation might prevent the acceleration of lung injury. Indeed, when large tidal volumes are delivered, this can lead to repeated over-distension of alveoli and further aggravate injury by volutrauma [6,35]. When lung protective ventilation is used, the aggravation of lung injury may depend on the degree of the reduction in aerated lung volume and the tidal volume used [36]. In the present study, the degree of the reduction in aerated lung volume following lung lavage was not severe as indicated by the mean P/F ratio above 400 on the high PEEP in experimental groups. In contrast, compared to healthy control group (HC), ARDS animals without suctioning demonstrated to have significant lung injury, which was accompanied by potential alteration of important inflammatory cytokines in the present study. However, despite these facts, we are not yet in a position to extrapolate the current findings with full confidence to the patients with established ARDS.

One notable and unique features of the present study is detection of crucial inflammatory cytokines related to ARDS in our experimental model, both at serum and pulmonary levels (lavage induced ARDS with surfactant depletion), i.e., both protein and mRNA expression. To date, no study using the similar experimental setting has performed such molecular analysis using repeated endotracheal suctioning. The potential inflammatory cytokines namely TNF-α and IL-6 were unchanged after 6 h of repeated endotracheal suctioning between the CS and OS groups at both the circulatory and pulmonary levels. The current finding is consistent with that of a recent study where oleic acid-induced ARDS model lacked significant changes in IL-6 and TNF-α at circulatory level in CS compared to OS [37]. However, unlike the present study, this previous study did not investigate levels of pulmonary cytokines, and, further, it only performed endotracheal suctioning once [37]. Thus, it seems that although the induction method of ARDS is different in our current study from that of Zhao F et al. [37] that used a different number of endotracheal suctioning protocols; the expression of serum IL-6 and TNF-α are essentially similar. Future studies should focus in depth on the changes of molecular pattern of potential inflammatory cytokines in these ARDS models with repeated endotracheal suctioning over a longer period of time. From the current findings, it can be stated that the number of endotracheal suctioning, whether repeated or single, may not affect circulatory levels of cytokines.

Repeated derecruitments induced by OS in our model did not exacerbate lung injury, based on the morphological as well as the molecular expression of crucial inflammatory cytokines compared to CS. However, this study demonstrated that CS prevents gradual reductions in arterial oxygenation, whereas the use of repeated OS caused progressive desaturation. Recently, patients undergoing mechanical ventilation are mananged according to lung-protective strategies in order to avoid high alveolar pressure using small tidal volumes and to keep alveoli open at end-expiratory level with sufficient PEEP [5,6,35]. With the increased use of high PEEP, when ventilator circuit is disconnected, patients can be exposed to the risk of sudden derecruitment and continuous desaturation that could be harmful to ARDS patients. Our findings suggest that routine use of CS is preferable, especially for the patients requiring high PEEP, to avoid gradual reductions in arterial oxygenation with the use of repeated OS.

## Limitations of this study

One of the notable limitations of the present study is that animals were received a muscle relaxant, which may have inhibited the animal’s efforts to maintain lung volume and altered regional differences in lung volume and ventilation. In addition, the fraction of inspired oxygen was set at 100%. Indeed, the rate of absorption of gas from an unventilated lung area increases with an increasing FIO_2_[38], thereby exacerbating desaturation. However, in clinical practice, we often need to use high F _IO2_ in patients with severe hypoxemia as well. In addition, it is interesting to note that despite this limitation, our data are consistent with those of previous studies [12,13,25-27], thus giving relevance and importance to the present data. In addition, due to technical limitations, we did not directly measure lung volume (with inductive plethysmography or magnetometers), nor did we measure lung compliance, and thus one could argue that the loss of lung volume induced by endotracheal suctioning is somewhat speculative. Although we cannot ignore this limitation, we believe, based on the literature discussed above [12] that the fall in oxygen saturation observed here might originate from alveolar derecruitment.

Secondly, we used the lavage-induced surfactant depleted ARDS models. Using this animal model, in which lung injury is induced by repeated broncho-alveolar saline lavage, response to repeated endotracheal suctioning can be more easily attained than in other animal models. More studies involving different animals with different endotracheal suctioning protocols using various models of lung injuries should be conducted before making clinical management recommendations that are applicable to patients with ARDS. In addition, the observation period in the present study still may be too short. Future studies should focus on examining the effects of repeated endotracheal suctioning over a longer period of time on the aggravation of lung injury in ARDS, which will more likely simulate the prevailing conditions in clinical settings.

## Conclusion

Repeated OS during mechanical ventilation does not exacerbate lung injury in the repeatedly derecruited lung over a long time (6 hours) by repeated endotracheal suctioning compared to CS based on both histological and molecular analyses. Arterial desaturation induced by repeated OS causes a gradual and time-dependent decline in ARDS during mechanical ventilation compared to CS and this finding makes the routine use of CS preferable, especially for the patients requiring high PEEP, to avoid gradual reductions in arterial oxygenation with the use of repeated OS.

## Key message

1) Arterial desaturation induced by repeated OS causes a gradual and time-dependent decline in ARDS during mechanical ventilation compared to CS.

2) Repeated OS during mechanical ventilation does not exacerbate lung injury in repeatedly derecruited lung over a long time (6 hours) by repeated endotracheal suctioning compared to CS based on both histological and molecular analyses.

3) Routine use of CS is preferable, especially for the patients requiring high PEEP, to avoid gradual reductions in arterial oxygenation with the use of repeated OS.

## Abbreviations

OS: Open endotracheal suctioning; CS: Closed endotracheal suctioning; HC: Healthy control; P/F ratio: PaO_2_/FIO_2_ratio; ARDS: Acute respiratory distress syndrome; PEEP: Positive end expiratory pressure; VILI: Ventilator–induced lung injury; PIP: Peak inspiratory pressure; IL-6: Interleukin-6; TNF-α: Tumor necrosis factor –alpha; GAPDH: Glyceraldehyde-3-phosphate dehydrogenase.

## Competing interests

None of the authors have any conflicts of interest associated with this study.

## Authors’ contributions

HS designed and performed the experiment, summarized and analyzed the data and wrote the manuscript. NS, SJ, JK, KM, MO, SK performed the experiment and summarized the data and edited the manuscript. SJ analyzed the data and edited the manuscript. TU, TM designed the experiment, analyzed the data and edited the manuscript. All authors read and approved the final manuscript.

## Pre-publication history

The pre-publication history for this paper can be accessed here:

http://www.biomedcentral.com/1471-2253/13/47/prepub
